# Induced pluripotent stem cell technology for modelling and therapy of cerebellar ataxia

**DOI:** 10.1098/rsob.150056

**Published:** 2015-07-01

**Authors:** Lauren M. Watson, Maggie M. K. Wong, Esther B. E. Becker

**Affiliations:** Department of Physiology, Anatomy and Genetics, University of Oxford, Oxford, UK

**Keywords:** induced pluripotent stem cells, cerebellum, ataxia, neurodegenerative disease

## Abstract

Induced pluripotent stem cell (iPSC) technology has emerged as an important tool in understanding, and potentially reversing, disease pathology. This is particularly true in the case of neurodegenerative diseases, in which the affected cell types are not readily accessible for study. Since the first descriptions of iPSC-based disease modelling, considerable advances have been made in understanding the aetiology and progression of a diverse array of neurodegenerative conditions, including Parkinson's disease and Alzheimer's disease. To date, however, relatively few studies have succeeded in using iPSCs to model the neurodegeneration observed in cerebellar ataxia. Given the distinct neurodevelopmental phenotypes associated with certain types of ataxia, iPSC-based models are likely to provide significant insights, not only into disease progression, but also to the development of early-intervention therapies. In this review, we describe the existing iPSC-based disease models of this heterogeneous group of conditions and explore the challenges associated with generating cerebellar neurons from iPSCs, which have thus far hindered the expansion of this research.

## 1. Introduction

The cerebellar ataxias are a diverse group of neurological disorders, defined by a loss of motor coordination which results from the degeneration of specific populations of neurons in the cerebellar cortex, brainstem and spinocerebellar tracts [1,2]. Although gait ataxia is the most common manifestation, non-ataxia symptoms, including oculomotor disturbances, dysarthria and extracerebellar symptoms, are often observed [3].

Cerebellar ataxia may arise either sporadically or as the result of an inherited genetic defect [1,2,4]. Non-genetic causes of ataxia include infections, alcoholism, vitamin deficiencies, immune-mediated or paraneoplastic diseases (reviewed in [1,5]). Genetic ataxias are usually classified according to their pattern of inheritance (autosomal dominant, recessive, X-linked or mitochondrial) and are highly heterogeneous, with over 60 subtypes identified to date [2,4]. Age of onset ranges from infancy to over 65 years, and depends largely on the mode of inheritance and the nature of the causative mutation, which includes trinucleotide or pentanucleotide repeats (coding or non-coding) and conventional mutations [6,7].

Although a vast number of causative genes have been identified, several common pathophysiological pathways appear to underlie the neurodegeneration observed in these conditions. These include mitochondrial defects, DNA repair deficiency and chaperone dysfunction in recessive ataxias, and transcriptional dysregulation, protein aggregation, ion channel defects, autophagy, mitochondrial defects and RNA toxicity in the case of dominant ataxias [8–10]. These pathways converge to cause cerebellar (and predominantly Purkinje cell) dysfunction [11]. Once thought of as purely degenerative conditions, mounting evidence now suggests that alterations in neuronal excitability and synaptic signalling, which precede neuropathology, may contribute to disease symptoms in a number of cerebellar ataxias [12]. These abnormalities may in turn be linked to defects in neuronal development and physiology, underscoring the need to understand the early processes that precede degeneration [13].

Treatment for the cerebellar ataxias is largely palliative. Even though clinical trials appear to be fairly advanced in some cases—in particular, Friedreich's ataxia (FRDA) [14]—no treatments have thus far been reported to slow progression or to ameliorate clinical symptoms. There is thus a pressing need for suitable, disease-relevant cell models in which to study disease progression and to screen potential therapies.

Rapid developments in the field of induced pluripotent stem cell (iPSC) technology offer the opportunity to combine the genetic accuracy of patient-derived cell models with the disease-relevance of specific cell types in the central nervous system (CNS). Since the first description of the use of reprogramming factors to generate stem cells from somatic cells [15–17], human iPSCs have been generated from a wide variety of easily accessible source tissues, including skin and blood cells [18–21]. Refinement of reprogramming methods now allows for iPSC generation without genomic integration of reprogramming factors, using expression plasmids, non-integrating viruses, recombinant proteins, small molecules and synthetically modified mRNAs or miRNAs (reviewed in [22]).

The potential of iPSCs to differentiate into any cell type of the body, offering the opportunity to study diseases of the CNS without the need for invasive surgical techniques, has been widely exploited, notably in the derivation of neurons from patients with a variety of neurodegenerative conditions [23–26]. Protocols have now been established for the differentiation of human iPSCs into cells with phenotypes resembling dopaminergic (DA), glutamatergic, GABAergic and motor neurons, as well as medium spiny neurons (MSNs) of the striatum, and glial progenitors (reviewed in [27]). Unlike patient post-mortem samples or animal models, iPSC-derived neurons allow for the study of specific neuronal subtypes at an early disease stage, without the need for exogenous overexpression of pathogenic proteins [28,29]. However, accurate recapitulation of disease-relevant tissues *in vitro* remains a challenge, requiring a precise understanding of the complex molecular events underpinning the development of each neuronal subtype, and an accurate set of criteria by which to identify and characterize the generated cells [27,30,31].

Although significant advances have been made, many protocols remain complex, requiring long periods of differentiation and expensive reagents, and yielding heterogeneous populations of neurons [27]. As a result, *in vitro* models of certain neuronal lineages—and, consequently, the diseases caused by their degeneration—have remained elusive. Here, we review the existing iPSC-based models of neurodegeneration, with a particular emphasis on the cerebellar ataxias, and explore the challenges associated with generating cerebellar neurons from iPSCs, which have thus far hindered the expansion of this research.

## 2. Use of induced pluripotent stem cells to model neurodegenerative diseases

The earliest reports of iPSC-based models of neurodegenerative disease detailed the generation of motor neurons from patients with inherited conditions, including amyotrophic lateral sclerosis (ALS) [25] and spinal muscular atrophy (SMA) [26]. Although these studies confirmed the potential for iPSC reprogramming and differentiation regardless of patient age or disease stage, reports of phenotypic severity were variable, raising concerns about the suitability of iPSC-based models to fully recapitulate late-onset conditions *in vitro*. Neurons derived from iPSCs have subsequently been used to model a variety of other neurodegenerative disorders, with Parkinson's disease (PD) [32,33], Alzheimer's disease (AD) [34–36] and Huntington's disease (HD) [24,37] being among the most extensively studied.

Differentiation of DA neurons from iPSCs has been demonstrated to be relatively robust and reproducible, allowing for the generation of neuronal models from patients carrying a variety of mutations in key genes implicated in familial PD, including *SNCA*, *PINK1*, *PARK2*, *GBA1* and *LRRK2* (reviewed in [27,38]). These neurons exhibited common signs of pathophysiology, such as enhanced susceptibility to oxidative stress, defects in the lysosomal and autophagic pathways, and altered calcium homeostasis. Notably, several of these defects could not be reproduced in fibroblasts taken from the same patients [39–41], highlighting the need for disease-relevant cell models of PD and other neurodegenerative diseases.

Similar to PD, the majority of AD cases are sporadic, rather than familial [29,35]. Several studies have successfully modelled the Mendelian forms of AD, generating neurons from patients with *APP*, *PSEN1* and *PSEN2* mutations, which exhibited phenotypes consistent with current hypotheses regarding AD pathogenesis (reviewed in [27,29]). Moreover, iPSC-based models offer the exciting possibility to study cells from patients with sporadic AD without prior knowledge of the causative genetic defects. The recent generation of iPSC-derived neurons from sporadic AD patients has allowed for the comparison of cellular phenotypes between the two forms of the disease, and for the identification of novel AD-associated networks of gene regulation [34,42,43].

HD, a dominantly inherited neurodegenerative disorder caused by mutations in the *HTT* gene, belongs to the family of polyglutamine (polyQ) diseases, which also includes a number of the dominant cerebellar ataxias [9]. HD has been extensively studied in stem-cell-based models, using established protocols for the differentiation of iPSCs into cells resembling MSNs, the cell type most affected by the disease (reviewed in [44]). A number of studies using iPSCs, neural stem cells and neurons derived from HD patients have demonstrated phenotypes including elevated lysosomal activity, mitochondrial deficits, alterations in gene expression patterns, as well as disease-associated changes in electrophysiology, cell adhesion, metabolism and cell death, many of which were CAG repeat-length-dependent [44,45]. Despite these findings, concerns remain regarding the correlation between iPSC-derived models of disease and native MSNs in the human brain, particularly regarding age and disease stage [44]. The requirement for treatment with a proteasomal stressor to induce the formation of Huntingtin protein aggregates (a hallmark of the disease in mouse models) in patient-derived stem cell models is just one example of the challenges associated with modelling late-onset disorders *in vitro* [46].

## 3. Induced pluripotent stem cells for modelling cerebellar ataxias

In contrast to the neurodegenerative diseases described above, relatively few studies have succeeded in generating iPSC-based models of the cerebellar ataxias. Of these, none have successfully recapitulated the cerebellar neuronal dysfunction and degeneration known to characterize these conditions.

### 3.1. Friedreich's ataxia

FRDA is the most common form of autosomal recessive ataxia, with a prevalence of 2–4.5 per 100 000. It is characterized by gait and limb ataxia, dysarthria and loss of tendon reflexes, with symptoms usually appearing before the age of 20 years. Distinct from many other cerebellar ataxias, FRDA primarily affects the peripheral sensory neurons (PSNs) and is considered a multi-system condition, with extra-neurological signs including diabetes and cardiomyopathy, the latter being the most common cause of death in patients [2,10].

FRDA is most commonly caused by a GAA-TTC trinucleotide repeat expansion in the first intron of the *FXN* gene, encoding the mitochondrial protein Frataxin [47]. The expansion results in epigenetic gene silencing and loss of Frataxin expression in affected individuals [48,49]. Although the function of Frataxin remains incompletely understood, evidence points to its involvement in mitochondrial iron–sulfur cluster biogenesis [50,51]. Frataxin deficiency in FRDA is believed to result in altered iron metabolism, impaired mitochondrial function and increased sensitivity to oxidative stress in cells of the dorsal root ganglia proprioceptive neurons and neurons of the deep cerebellar nuclei, among others [52].

The mechanism by which Frataxin, a ubiquitously expressed protein, causes cell-type-specific pathologies has been partly attributed to the genetic basis of FRDA, with the GAA-TTC repeat showing tissue-specific and age-related somatic instability [53]. Much effort has gone into developing relevant cellular models of FRDA, as available animal models fail to fully recapitulate the cellular features of pathology [53–55].

Several groups have succeeded in generating iPSC lines from patients with FRDA [56–60]. In all cases, patient iPSCs maintained Frataxin gene repression and displayed GAA-TTC repeat instability similar to that seen in patient somatic cells, although no biochemical phenotype was observed in the stem cells themselves. This repeat instability, which was not observed in patient fibroblasts or neuronal stem cells, correlates with the expression of mismatch repair enzymes such as Msh2, which are highly active in pluripotent cells, and have been shown to occupy intron 1 of the *FXN* gene in iPSCs [57,60].

Early studies of FRDA iPSC-derived cardiomyocytes and generic neuronal populations showed similar disease-relevant reductions in Frataxin mRNA and protein levels, as well as signs of mitochondrial functional impairment [58,59]. More recently, Eigentler *et al*. [56] applied a development-based differentiation protocol to specifically generate PSNs, one of the affected cell types in FRDA. By closely monitoring the progress of the cells through various stages of *in vitro* differentiation, they were able to evaluate the hypothesis that FRDA pathology begins early in development [61,62]. Importantly, they identified impaired upregulation of Frataxin during the differentiation of PSNs from FRDA iPSCs, in addition to the Frataxin expression deficits observed in FRDA iPSCs and neural crest cells in previous studies [57–60]. These results suggest strongly that a selective deficiency in Frataxin may have detrimental early effects in PSN development, which accumulate over time to result in cell-type-specific degradation [56].

Although these studies have provided insights into the pathogenesis of FRDA in cardiomyocytes and PSNs, additional work is required to elucidate the role of Frataxin deficits in other affected cell types, such as the neurons of the deep cerebellar nuclei. Of particular interest in future research will be the comparison between affected and unaffected neuronal types, in order to identify particular characteristics that render specific neuronal populations vulnerable to a genetic insult present in all cells.

### 3.2. Spinocerebellar ataxias

Although studies of FRDA were among the first to demonstrate a consistent, disease-relevant cellular phenotype in iPSC-derived human cells obtained from ataxia patients, it should be noted that all of the abovementioned results were generated in cell models of the extracerebellar sites of disease. Generation of *in vitro* models of the dominantly inherited cerebellar ataxias, whose pathologies centre on cerebellar (and particularly, Purkinje cell) degeneration, has proved to be significantly more challenging.

A handful of studies published to date have focused on the polyglutamine spinocerebellar ataxias (SCAs). All of these have been based on generic differentiation towards the neural lineage, as opposed to the generation of specific neuronal subtypes, with often inconsistent reports of modest cellular phenotypes. Xia *et al*. [63] generated iPSC lines from one SCA2 patient and an unaffected control. They observed abnormalities in neural rosette formation during *in vitro* differentiation from SCA2 iPSCs, although patient cells ultimately differentiated successfully into neural stem cells and neurofilament H-positive neurons. Whereas patient and control fibroblasts showed comparable levels of expression of the disease-causing protein Ataxin-2, this expression was decreased in SCA2 neural stem cells. Time-lapse imaging also indicated that SCA2 neural cells were short-lived, compared with controls [63]. Of note, the CAG repeat expansion in Ataxin-2 remained stable throughout reprogramming and differentiation, a feature common to polyglutamine diseases, which distinguishes them from FRDA [23,64]. The generation of iPSCs and βIII tubulin-positive neurons from a single SCA7 patient has also been reported, although that study did not investigate a phenotype in these cells [65].

Perhaps the most promising research to date has been the generation of βIII tubulin-positive neurons from SCA3 patient iPSCs, first reported in 2011 by Koch *et al*. [23]. In response to l-glutamate-induced excitation, these neurons underwent calcium-dependent proteolysis of Ataxin-3, the disease-causing protein, triggering its aggregation—a hallmark of the disease in patients. This aggregation, which was also found to depend on functional sodium and potassium channels, as well as ionotropic and voltage-gated calcium channels, was abolished by calpain inhibition, confirming the key role of this protease in Ataxin-3 cleavage. Furthermore, aggregation was not observed in iPSCs, fibroblasts or glia generated by similar protocols, providing a possible explanation for the neuron-specific phenotype observed in SCA3 patients.

While these studies have provided some insights into the pathogenesis of the SCAs, several shortcomings remain to be addressed. To date, research has focused on the polyglutamine SCAs. It would be useful to develop models of other classes of SCA in the future, in order to gain further insights into the pathology of these conditions. It is also likely that more accurate phenotypes may be identified through the use of larger patient cohorts, which are more representative of the spectrum of disease. Most important, however, is the need for models of the specific neuronal subtypes most prone to dysfunction in ataxia—those of the cerebellum.

## 4. Generating cerebellar neurons using induced pluripotent stem cells

The human cerebellum contains more neurons than the entire cerebral cortex. It is one of the most regularly organized CNS structures, composed of eight main cell types arranged in repeated structures of well-defined layers [66]. Despite significant advances in the understanding of cerebellar circuitry and microphysiology, a number of challenges have hindered the establishment of iPSC-based models of cerebellar degeneration. Chief among these is the need to recapitulate the complex *in vivo* events underlying cerebellar development and neuronal lineage specification *in vitro*.

### 4.1. Cerebellar development

Cerebellar development *in vivo* may be broadly divided into three phases: specification of the cerebellar territory, or ‘anlage’, during early hindbrain development; allocation of the various cerebellar cell types along the dorsoventral axis; and transit amplification and migration of granule cell precursors from the external granule layer over the cerebellar surface to form the internal granule layer [67,68].

**Figure 1.**
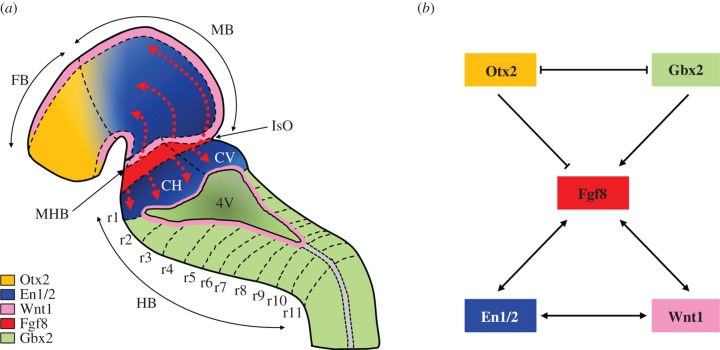
Early specification of the cerebellar anlage. (*a*) Colour-coded distribution of the main gene expression gradients within the mouse neural tube at E11.5. The spatio-temporal interactions between the expression gradients define the boundaries between the forebrain (FB), midbrain (MB) and hindbrain (HB). The reciprocal inhibition between Otx2 and Gbx2 positions the isthmic organizer (IsO) at the midbrain–hindbrain boundary (MHB). The IsO is critical for the induction of the cerebellum from rhombomeres r0 and r1 via expression of Fgf8. Red dotted arrows indicate the rostral and caudal gradients of Fgf8 expression. The roofplate of the fourth ventricle (4V) is indicated. CH, cerebellar hemisphere; CV, cerebellar vermis. Adapted from [67]. (*b*) Genetic interactions between the major genes that are required for the coordinated patterning of the midbrain/hindbrain region.

During early development, the neural tube is progressively regionalized, under the control of temporally and spatially coordinated gradients of gene expression [67] (figure 1). The anterior limit of the cerebellum is defined by the midbrain–hindbrain boundary (MHB) through signalling from a region of the neuroepithelium known as the isthmic organizer (IsO), which provides structural polarity to the surrounding regions [67,69]. At a molecular level, the MHB is maintained by differential expression of Otx2 in the rostral neuroepithelium, and Gbx2 in the posterior domain. The reciprocal inhibition of these genes, further stabilized by the expression of Fgf8 and Wnt1 at the IsO, leads to the formation of a sharp boundary between their expression domains, establishing the MHB, and marking the anterior border of the mature cerebellum [67,69].

**Figure 2.**
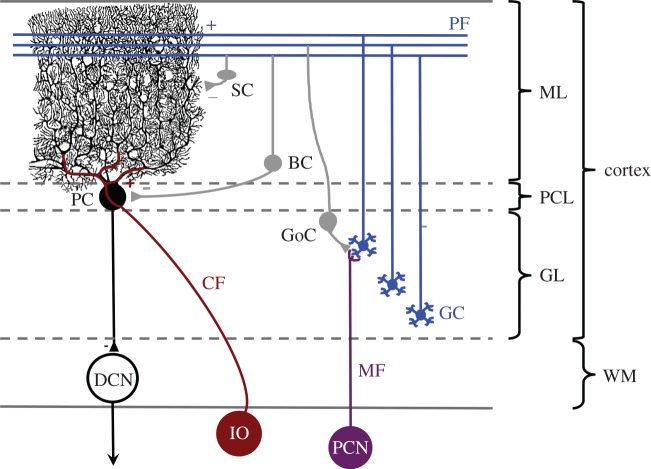
Schematic of the major cell types and circuitry of the cerebellum. Purkinje cells (PC) form the sole output of the cerebellar cortex and receive excitatory input from climbing fibres (CF) originating in the inferior olive (IO), and parallel fibres (PF), the axons of the granule cells (GC). GCs receive input from the mossy fibres (MF) coming from the precerebellar nuclei (PCN). Excitatory (+) and inhibitory (−) inputs are indicated. Minor cerebellar cell types including unipolar brush cells and Lugaro cells are not depicted. BC, basket cell; DCN, deep cerebellar nuclei; GL, granular layer; GoC, Golgi cell; ML, molecular layer; PCL, Purkinje cell layer; SC, stellate cell; WM, white matter. Reproduced from [70] with permission.

Cerebellar neurons arise from two germinal centres—the rhombic lip (the dorsal-most subdivision of the hindbrain neuroepithelium) and the ventricular zone [67,69]. The rhombic lip is responsible for the generation of cerebellar glutamatergic neurons including granule cells, unipolar brush cells and projection neurons of the deep cerebellar nuclei. By contrast, the ventricular zone gives rise to cerebellar GABAergic neurons (Purkinje, Lugaro, Golgi, basket and stellate cells), as well as Bergmann glia, which provide structural support for neuronal migration during development [67]. The most prominent cell type of the cerebellum is the Purkinje cell, with its soma located in the middle cortical layer (known as the Purkinje cell layer), and an extensive, flattened dendritic tree projecting into the molecular layer (figure 2). The dendrites of Purkinje cells receive synaptic input from parallel fibres of granule cells, the main excitatory interneurons of the cerebellar cortex. In mice, each parallel fibre makes contact with the dendrites of a few hundred Purkinje cells, while each Purkinje cell receives synaptic input from up to 200 000 parallel fibres. Purkinje cells provide the sole output from the cerebellar cortex, via a single inhibitory axon projected to one of the cerebellar nuclei [66].

### 4.2. Protocols for cerebellar neuronal differentiation from pluripotent stem cells

The complexity of cerebellar neuronal development, involving two germinal zones, and resulting in highly specialized cellular structures, presents a challenge to the development of effective *in vitro* differentiation protocols. This is particularly true for the Purkinje cells, whose massive size, morphological complexity and distinct electrophysiological properties render them particularly vulnerable to proteostatic insults and disturbances in ion channel function caused by mutations in ataxia disease genes [11].

The earliest studies of cerebellar differentiation from pluripotent cells sought to investigate whether local signals responsible for the specification of neural fates *in vivo* could be harnessed to drive differentiation of mouse embryonic stem cells (ESCs) *in vitro* into granule cells, Purkinje cells and cerebellar glial cells [71,72]. This was accomplished by sequential treatment of ESCs grown in suspension with secreted factors (Fgf8, Wnt1 and retinoic acid) known to initiate patterning in the cerebellar region of the neural tube; bone morphogenic proteins (Bmp6, Bmp7 and Gdf7), which induce early granule cell progenitor markers (such as Math1, Meis1 and Zic1); and mitogens (Shh and Jagged1), which control proliferation and induce the expression of granule cell markers (Pax2 and Pax6) [71,72]. Granule cell precursors were sorted by means of flow cytometry, and co-cultured with either postnatal mouse cerebellar neurons [72] or glial-conditioned medium [71], to promote maturation [71,72]. While these protocols succeeded in generating granule cells at a relatively high efficiency, the proportion of Purkinje cells in the resulting cultures was extremely low (0.5–3% of total neurons, even after the addition of Fgf8, which was shown to exhibit enhancing effects) [72]. These results have subsequently been replicated in human ESCs using a similar approach, by subjecting cells to a series of inductive signals and differentiation factors designed to mimic the early patterning of the cerebellum [73]. The result was a mixed population of neurons—approximately 61% glutamatergic, 36% GABA-positive and 41% positive for the granule cell marker Girk2. The proportion of total cells expressing early Purkinje cell markers was significantly higher than in earlier reports involving mouse ESCs (11% L7-positive, 29% Gad67-positive and 13% Lhx5-positive), although still relatively low. This is not entirely unexpected, given the relatively small proportion of Purkinje cells in the total volume of the cerebellum. Importantly, however, this study failed to generate neurons with a convincing Purkinje cell morphology. In addition, the cost and variability associated with sequential treatment with such large numbers of growth factors has meant that this work is yet to be replicated.

In an effort to improve the efficiency of mouse Purkinje cell generation, survival and maturation *in vitro*, Tao *et al*. [74] proposed a system of co-culture using a feeder layer of dissociated cerebellar cells prepared from mice at postnatal day 6–8, or organotypic slice cultures of whole mouse cerebellum. The result was a twofold to ninefold increase in numbers of surviving Purkinje cells. Both co-cultured and feeder-free Purkinje cells showed equivalent maturation after 28 days in culture, with parameters including dendrite and soma area, maximum dendrite length and number of branching points approximating those of neonatal Purkinje cells [74].

Muguruma *et al*. [75] explored a different strategy, focusing on recapitulating the endogenous self-inductive signalling microenvironments associated with the IsO, using mouse ESCs. They employed a combination of just three factors—insulin, which exhibits moderate caudalizing activity; Fgf2, which has been shown to induce rhombomere 1; and cyclopamine, a Hedgehog receptor antagonist, which promotes dorsalization indirectly by inhibiting the ventralizing effect of endogenous Shh. This approach greatly enhanced Purkinje cell differentiation efficiency in cell aggregates cultured in suspension (35–42% of total cells expressing early Purkinje cell markers Corl2 and L7 by day 11 of differentiation). To further improve the yield of Purkinje cells, cells positive for Neph3 (also known as Kirrel2, a cell-surface marker upregulated by Ptf1a, a transcription factor essential for Purkinje cell generation) were purified by fluorescence-activated cell sorting (FACS) and co-cultured with mouse cerebellar granule cells. The resultant neuronal population consisted of 82.7% Corl2-positive cells, which developed highly arborized dendrites, and went on to express mature Purkinje cell markers such as the glutamate receptor subunit GluRδ2 [75]. This three-step protocol, involving MHB proximity specification, dorsal plate specification (through the generation of Neph3-positive cerebellar plate progenitors) and Purkinje cell differentiation from Neph3-positive progenitors, thus provides a significant improvement (up to 30-fold) over previous reports of the frequency of Purkinje cell differentiation. One possible explanation is that the combination of two weak caudalizing signals (Fgf2 and insulin) is sufficient to pre-pattern the pluripotent cell aggregates to a broad midbrain–hindbrain fate, without interfering with the endogenous self-inductive programme, which is usually controlled by signals from the IsO [75]. Based on this rationale, it is plausible that the direct application of exogenous Fgf8 and Wnt1 employed by earlier studies could have disrupted endogenous signalling pathways, resulting in relatively less efficient differentiation.

**Figure 3.**
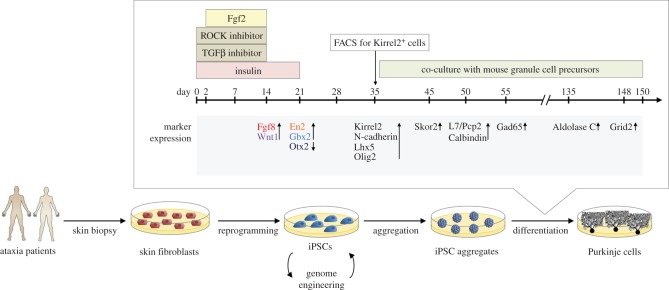
Schematic of the proposed differentiation of Purkinje cells from iPSCs obtained from ataxia patients, through the recapitulation of endogenous self-inductive signalling. The depicted protocol is based on the method described for the cerebellar differentiation from hESCs, and was adapted from [76]. Skin fibroblasts obtained from ataxia patients are reprogrammed into iPSCs. These might be subjected to genome engineering to repair or induce ataxia-causing gene mutations. iPSCs are then aggregated in suspension culture, with aggregates subsequently undergoing differentiation into mature Purkinje neurons following the depicted 150-day protocol. Day 0 corresponds to the aggregation of iPSCs. Insulin, Rho kinase (ROCK) inhibitor, TGFβ inhibitor and Fgf2 are added to promote the self-inductive signalling in the indicated period. Kirrel2-positive Purkinje cells are enriched using FACS on day 35 and co-cultured with mouse granule cell precursors until mature (day 150). Up- and downregulated expression of markers for neural precursors and Purkinje cells are indicated.

These results have subsequently been replicated using human ESCs with some minor modifications, including the addition of the TGFβ inhibitor SB431542 to the culture, which inhibits mesenchymal differentiation and promotes differentiation towards a neuroectodermal fate [76]. These cells demonstrated robust neuronal differentiation, expressing midbrain–hindbrain markers such as En2 and Gbx2 after 14–21 days in culture. By 35 days in culture, approximately 28% of cells expressed the Purkinje cell progenitor marker Neph3/Kirrel2 and were subsequently purified by FACS, using an anti-Kirrel2 antibody. Ten days after FACS, 44.7% of Kirrel2-positive cells were found to express the Purkinje cell marker Skor2. Following long-term co-culture with mouse cerebellar granule cell precursors, these neurons expressed the mature Purkinje cell markers L7, Calbindin, Aldolase C and Lhx5, and exhibited morphology and electrophysiology comparable with that of Purkinje cells *in vivo* [76]. It is conceivable that a similar strategy might be applied to differentiate human iPSCs into Purkinje cells (figure 3). The same study also reported the generation of granule cells from human ESCs via Atoh1-positive progenitors at a slightly lower efficiency (18% of total cells at day 35). Interestingly, the addition of Fgf19 and Sdf1 during differentiation promoted the spontaneous generation of polarized neural tube-like structures with a three-layer cytoarchitecture reminiscent of the embryonic cerebellum, suggesting that human ESC-derived cerebellar progenitors show significant potential for self-organization [76].

A single study has thus far reported replication of these findings using human iPSCs (as opposed to ESCs) [77]. However, the controversial method proposed for enhancing Purkinje cell maturation, involving co-culture with human fetal cerebellar slices, suggests that more work may be needed to optimize these culture systems before they can be used routinely for disease modelling and cell-replacement therapies.

### 4.3. Neurodevelopmental aspects of cerebellar ataxia

The suitability of iPSC-derived neurons for modelling late-onset conditions remains controversial [78], particularly given the immature, fetal nature of the neurons generated from these cells [79]. It is possible that the phenotypes of adult-onset conditions, such as PD and ALS, may never be fully recapitulated under basal cell culture conditions, but may require the addition of neural stressors such as reactive oxygen species, pro-inflammatory factors or even toxins in order to manifest [33,80,81]. Similar concerns might be raised regarding cellular models of the cerebellar ataxias. However, mounting evidence from cell and animal models indicates that abnormalities in early Purkinje cell development may contribute to the pathogenesis of the ataxias, suggesting a vital role for iPSC-based modelling in unravelling the neurodevelopmental aspects of these diseases [13].

One such example is the SCA1 transgenic mouse model, which exhibits a much milder disease phenotype when expression of mutant Ataxin-1 is delayed until after cerebellar maturation, suggesting a role for the disease-causing protein Ataxin-1 in Purkinje cell development [82]. Indeed, Ataxin-1, as well as another ataxia-associated protein, Ataxin-3, have been linked to the retinoic acid-related orphan nuclear receptor alpha (RORα) pathway, which is critical for cerebellar development [82,83]. Mouse models expressing mutant forms of either of these disease-associated genes show reduced levels of RORα, together with abnormalities in Purkinje cell development, including impaired dendritic arborization and pruning [83–86].

Similar impairments in Purkinje cell dendritogenesis and synapse formation have been described in *staggerer* mice harbouring recessive mutations in the *RORA* gene, encoding RORα [87], as well as in mouse models of SCA5 [88], and in cell and mouse models of SCA14 [89–91]. In addition, genetic mouse mutants such as the *Moonwalker* (*Mwk*) mouse, which harbours a dominant gain-of-function mutation in the TRPC3 ion channel, have confirmed the relationship between abnormal Purkinje cell development and ataxia [92]. This abnormal development is thought to arise via disruption of the metabotropic glutamate receptor subtype 1 (mGluR1) signalling pathway, in which TRPC3 is involved [13,93]. mGluR1 signalling, which plays a vital role in Purkinje cell function [94], has been shown to be disrupted in a number of other mouse models of ataxia, including SCA1 [87,95], SCA3 [83] and SCA5 [96]. Given the increasing evidence for Purkinje cell developmental abnormalities in cerebellar ataxia, it seems likely that iPSC-derived models, which are capable of recapitulating early developmental events *in vitro*, will be invaluable in unravelling the pathogenic complexities of these conditions.

A number of challenges remain to be addressed in developing iPSC-derived cerebellar neurons. In addition to those described above, the low prevalence of the cerebellar ataxias in the general population has meant that most studies have been based on results from far smaller cohorts of patients than those investigating PD or AD. Nonetheless, the establishment of efficient, reproducible cellular models of cerebellar Purkinje cell dysfunction and degeneration *in vitro* will be important not only in elucidating the molecular basis of these diseases, but also in the development of effective therapies.

## 5. Therapeutic applications

### 5.1. Modelling and drug screening

Perhaps the most straightforward application of iPSC models for therapeutic development is as a tool for drug target identification and screening. Few effective drugs exist for the treatment of neurodegenerative diseases. Although animal models are frequently used to evaluate the safety and efficacy of novel therapies, numerous challenges, including species-specific differences in gene expression, as well as their lack of suitability for high-throughput screening, make the use of such models less than ideal [79]. By comparison, iPSCs and their derivatives offer an unlimited source of disease-relevant cells, allowing access to otherwise inaccessible human tissues and enabling repeated experiments on a large scale at a stage approximating early development, well before clinical symptoms emerge [97,98]. These cells may be used for toxicity screens, to assess the effects of novel compounds on relevant cell types, or for differentiation screens, to identify compounds capable of enhancing self-renewal, maturation or survival [79]. Establishment and differentiation of iPSC lines from patients carrying disease mutations, such as Purkinje cells from patients with cerebellar ataxia, provides a model in which to screen for drugs that may correct the observed disease phenotype, an approach already used with some success in models of SMA and familial dysautonomia [79]. These models may also be useful in identifying novel downstream targets of disease genes [99], as well as stressors capable of eliciting phenotypes in late-onset conditions [100], and genotypic modifiers of disease progression and drug response [38,100,101]. Indeed, high-throughput stem-cell-based screening has been used to identify small molecules capable of enhancing the survival of motor neurons or inhibiting microglial neurotoxicity [102,103]. Recent studies have also evaluated the efficacy of therapeutic compounds in iPSC-based models of dry age-related macular degeneration and inherited PD, revealing in the latter case unique biomarker signatures which are likely to more accurately reflect the drug response in human patients [104,105].

Despite these promising results, a number of issues remain to be addressed before iPSC-based preclinical screens are widely adopted. One of the most crucial is the need to establish a reliable, consistent disease phenotype in a cell type that can be reproducibly generated in relatively large quantities *in vitro* [79].

### 5.2. Promises and challenges of genome editing technologies

Investigating the causative role of a particular mutation is often confounded by the effects of inter-individual genetic variation. One method of bypassing these effects involves the comparison of control and patient cell lines with identical genetic backgrounds, generated by means of genetic manipulation. This is typically accomplished through the introduction or correction of disease-causing mutations into cells, which can then be used either to create new disease models or to confirm a causative role for a particular mutation [79,106,107]. The value of genome editing to correct mutations and effect phenotypic rescue *in vitro* has been demonstrated in models of HD [107], SMN [108], tauopathy [109], myotonic dystrophy [110] and PD [99], among others [111,112]. Most studies to date have employed a zinc finger nuclease or TAL effector nuclease-mediated approach, both of which require substantial protein engineering for each new DNA target site [113]. However, the rapid development of CRISPR/Cas9-mediated genome editing, a simple two-component system involving a single guide RNA capable of directing the Cas9 endonuclease to effect double stranded DNA breaks at multiple target sites with only minor alterations in RNA sequence, is likely to result in significant advances in the field [113]. Indeed, many of the concerns initially raised regarding this technique, including off-target effects and relatively low efficiencies of mutation correction by homology-directed recombination, have rapidly been addressed [114,115].

### 5.3. Transplantation of stem cells

Differentiating iPSCs into disease-relevant tissues offers an opportunity for cell-replacement therapies for intractable diseases. Autologous transplantation of patient-specific iPSC-derived neurons in particular circumvents many of the current limitations associated with allogeneic transplants for neurodegenerative disease, including immunogenicity and the need for fetal donors [116]. This approach has shown significant promise in mouse, rat and primate models of PD, where transplantation of iPSC-derived, mutation-corrected DA neurons was well tolerated for up to two years post-transplant, surviving and engrafting into the site of transplant and, in some cases, leading to moderate phenotypic reversal [27,116,117].

Transplantation and functional integration of stem-cell-derived neural cells has also been assessed in the cerebellum. Cell survival rate following implantation into mouse neonatal cerebellum was typically low (0.1–0.3% of total injected cells), particularly in early studies, although those cells that survived showed characteristic granule cell morphology [71,72]. By contrast, Muguruma *et al*. [75] demonstrated efficient orthotropic integration of ESC-derived Purkinje cells and subsequent formation of normal synaptic connections to deep cerebellar nuclei targets. Before such approaches can be applied to the cerebellar ataxias, and neurodegenerative diseases in general, however, several challenges must be addressed, including the requirement for consistent protocols capable of generating large quantities of neurons in a homogeneous, xeno-free culture, in order to minimize the potential for tumourigenic or immunogenic effects [116]. The age at transplantation is also likely to be a key consideration, as integration efficiency has been shown to decrease dramatically in the postnatal (as opposed to fetal) brain [75].

## 6. Conclusion

IPSC-based models offer distinct advantages in the study of neurodegenerative diseases, including the inherited and acquired cerebellar ataxias. Cerebellar neuronal models are likely to provide valuable insights into the selective vulnerability of distinct neuronal subtypes in these conditions, particularly the Purkinje cells. To date, however, challenges associated with recapitulating the complex pathways underlying cerebellar development have hindered the development of efficient, reproducible protocols capable of producing sufficient numbers of these cells. The advent of novel approaches, such as the induction of IsO signalling *in vitro*, will hopefully circumvent these difficulties, allowing for the development of accurate, disease-relevant models for the study of the molecular mechanisms underlying cerebellar ataxia, and the development of long-awaited therapies.
